# Editorial: New tools and molecular advances in hyperproliferative skin disorders

**DOI:** 10.3389/fmed.2022.1002872

**Published:** 2022-08-11

**Authors:** Marika Quadri, Cristina Pellegrini, Tatiana Efimova, Elisabetta Palazzo

**Affiliations:** ^1^DermoLAB, Department of Surgical, Medical, Dental, and Morphological Science, University of Modena and Reggio Emilia, Modena, Italy; ^2^Dermatology, Department of Biotechnological and Applied Clinical Sciences, University of L'Aquila, L'Aquila, Italy; ^3^Department of Anatomy and Cell Biology, The George Washington University School of Medicine and Health Sciences, Washington, DC, United States; ^4^The George Washington Cancer Center, The George Washington University School of Medicine and Health Sciences, Washington, DC, United States; ^5^Department of Dermatology, The George Washington University School of Medicine and Health Sciences, Washington, DC, United States

**Keywords:** cSCC, psoriasis, atopic dermatitis, cancer models, PI3K/AKT, miRNA, AGEs

Hyperproliferative skin disorders (HSD) consist of various types of pathologies that present different skin manifestations in terms of severity and distribution of injuries (1, 2). They are commonly characterized by altered epidermal homeostasis causing uncontrolled skin proliferation and dysregulated differentiation. Psoriasis is a chronic immune-mediated HSD with both environmental and genetic components, in which the CD4^+^ T cell dysfunction seems to have a prominent role (3, 4). Its pathogenesis is not entirely understood and is subject to intensive investigation. Atopic dermatitis (AD) is a highly pruritic, chronic, multifactorial HSD involving the development of immune components, with a prevalence of Th2-responses in the acute phase and important epidermal barrier defects (5). The incidence of AD is increasing in developed countries, and the identification of risk factors and pathogenetic mechanisms is of great importance (6, 7). NMSC represents one of the most common cancers in the world, with a rising incidence every year. The two most common NMSCs are basal cell carcinoma (BCC) and cutaneous squamous cell carcinoma (cSCC), which arise from epidermal keratinocytes of the basal or squamous layers, respectively (8–10). Several tumor suppressor genes and proto-oncogenes have been identified as associated with the development of such lesions (11, 12).

Given the high heterogeneity of the HSD conditions, basic and translational research is essential for developing novel therapeutic strategies. The present Research Topic, “*New tools and molecular advances in hyperproliferative skin disorders*,” reports manuscripts by several scientists operating in the field, aiming to decipher the complexity of these diseases.

Mercurio et al. provide an extensive overview of the role and function of the PI3K/AKT/mTOR molecular axis in the pathogenesis of different HSD. They discuss recent evidence of the involvement of the PI3K/AKT/mTOR pathway in the development and progression of NMSC, enhancing cell proliferation and resistance to apoptosis. In inflammatory skin conditions, such as psoriasis and AD, the pathway shows a dual effect. In psoriatic lesions, the hyperactivation PI3K/AKT pathway could be implicated in regulating the senescent-like phenotype of epidermal keratinocytes, thus promoting a keratinocyte growth arrest. In AD, the pathway is involved in epidermal stratification, cornification, and immune-mediated inflammatory response. Finally, the authors summarize the current and future strategies targeting AKT/mTOR pathway.

Among HSD, psoriasis plays a prominent role because of its complexity. Recent evidence shows altered microRNA (miRNA) expression profiles in the psoriasis (13, 14). Circulating miRNAs in a patient's sera appear to correlate with the Psoriasis Area Severity Index (PASI) scores and may serve as biomarkers for diagnosis, progression, and therapy evaluation (15). In this issue, Xiuli and Honglin reviewed the dysregulation of miRNAs, focusing on two hallmarks of psoriasis: keratinocyte hyperproliferation and T-cells deregulation. They identified the existence of an up and down of miRNAs expressions forming distinct networks that collaborate to promote the development and maintenance of psoriasis by NF-kB, Notch, PTEN/PI3K/KT3, and STAT3 signaling pathways.

In the context of psoriasis, a mention should be made of those patients who develop psoriatic arthritis (PsA) after psoriasis or concomitantly with it. Among treatments, methotrexate (MTX) has greater efficacy in patients without arthritis and is less associated with hepatoxicity (16). Therefore, it is critical to identify biomarkers for predicting the development of PsA and the efficacy and hepatotoxicity of MTX. Methylenetetrahydrofolate reductase (MTHFR) is a crucial enzyme in homocysteine/methionine metabolism, and MTHFR polymorphisms have been associated with psoriasis risk (17). However, a link to PsA risk in the Chinese population is missing. Zhu et al. demonstrated that specific MTHFR SNPs (e.g., rs1801133 CC) could be associated with the development of PsA. The authors also explained that the MTX-related hepatotoxicity could depend on particular genotypes. Even though these results are a good starting point, they necessitate further investigation to increase the robustness of their data by increasing the size of the analyzed samples.

Advanced glycation end products (AGEs) accumulate in organs and tissues with aging and are linked to the pathogenesis of a multitude of age-related and chronic inflammatory diseases, such as diabetes, cardiovascular and neurodegenerative diseases, and renal failure (18, 19). In their comprehensive review, Chen et al. discuss recent literature on the role of AGEs in the skin. They highlight the biochemical pathways leading to AGEs formation and accumulation, provide a classification of AGEs found in the skin, and describe endogenous and exogenous sources of AGEs. The authors then summarize the effects of AGEs on the structure and functions of the epidermal and dermal compartments of the skin and the current information on the underlying molecular mechanisms by which AGEs cause damage in different skin layers. Moreover, the review also includes a comparative overview of several novel comprehensive methodologies for measuring the AGEs content in human skin. Finally, the mechanisms of the prevention and inhibition of the formation and accumulation of AGEs are discussed, suggesting that AGEs inhibitors could be of potential therapeutic benefit for treating skin diseases, promoting cutaneous wound healing, and delaying skin aging.

cSCC is a complex disease representing the second most common type of skin cancer (8, 10, 20). In recent years, several efforts have been made to understand the molecular processes underlying cSCC pathogenesis. Quadri et al. provided an update about the most important *in vitro* and *in vivo* tools developed for cSCC studies. They explained the use of two-dimensional (2D) cell culture and of the most recently established three-dimensional (3D) cSCC models (spheroids, organoids, and skin reconstruct), emphasizing their contributions to novel drug testing and pharmaceutical development. Moreover, the authors explained the continued necessity of using animal models in cancer research, which compensates for the lack of systemic components of *in vitro* models. The new zebrafish xenotransplant model allows monitoring tumor metastasis in real time. In the last years, several categories of cSCC-mouse models have been developed, which have been fundamental to understanding new molecular mechanisms underlying cSCC pathogenesis and evaluating drug response in an *in vivo* setting.

In summary, this Research Topic highlights the latest efforts in HSD research, focusing on identifying novel molecular networks, biomarkers, or signaling pathways and developing novel models that allow a better understanding of these skin conditions.

## Author contributions

All authors contribute to writing the manuscript and approved it for publication.

## Conflict of interest

The authors declare that the research was conducted in the absence of any commercial or financial relationships that could be construed as a potential conflict of interest.

## Publisher's note

All claims expressed in this article are solely those of the authors and do not necessarily represent those of their affiliated organizations, or those of the publisher, the editors and the reviewers. Any product that may be evaluated in this article, or claim that may be made by its manufacturer, is not guaranteed or endorsed by the publisher.
